# Shoulder injuries in polytraumatized patients: an analysis of the TraumaRegister DGU®

**DOI:** 10.1007/s00068-020-01340-1

**Published:** 2020-03-27

**Authors:** Thorben Briese, Christina Theisen, Benedikt Schliemann, Michael J. Raschke, Rolf Lefering, Andre Weimann

**Affiliations:** 1 Department of Trauma, Hand and Reconstructive Surgery, University Hospital Muenster, Albert-Schweitzer-Campus, Building W1, 48149 Muenster, Germany; 2 Department for Orthopedic Surgery, Schoen Clinic Duesseldorf, Am Heerdter Krankenhaus 2, 40549 Duesseldorf, Germany; 3 IFOM, Institute for Research in Operative Medicine, University of Witten, Herdecke, Germany; 4 OCP–Muenster, Orthopedic, Trauma and Hand Surgery, Schaumburgstrasse 1, 48145 Muenster, Germany

**Keywords:** Polytrauma, Shoulder injuries, Epidemiology, Injury severity score, Thoracic trauma, Traffic injury

## Abstract

**Background:**

The aim of the present study was to analyze the prevalence, epidemiology and relevance of shoulder injuries in polytraumatized patients in a large national trauma database.

We hypothesize a high prevalence of shoulder injuries in traffic accidents and a high prevalence of concomitant injuries of the thorax leading to an aggravated clinical course and higher Injury Severity Score (ISS). Furthermore, we hypothesize an increased rate of surgical treatment with the severity of the injury.

**Materials and methods:**

The retrospective analysis is based on the database (2002–2013) of the TraumaRegister DGU® and includes statistical data from 608 hospitals. The severity of injuries and trauma were scaled using the Abbreviated Injury Scale (AIS), and the Injury Severity Score (ISS), respectively. Patients with an ISS ≥ 16 were included in the study, and injuries were subdivided according to their anatomical involvement and analyzed with respect to the trauma mechanism and the resulting injuries.

**Results:**

In this study, 54,076 cases of patients with an ISS ≥ 16 were analyzed. Shoulder injuries occurred in 15,115 patients (27.9%). Of these, 68.5% were caused by traffic accidents, especially in motorbike, bicycle, and pedestrian accidents. We found more shoulder injuries in blunt trauma mechanisms. Moreover, patients with shoulder injuries spent on average 1.7 more days on the intensive care unit (ICU), or intermediate care unit (IMCU), according to the severity of the injury, and had longer overall hospital stays (26.2 vs. 24.1 days) than patients without shoulder injuries. The overall ISS was increased in patients with shoulder injuries, whereas an increase of mortality could not be identified. Concomitant thoracic injuries occurred significantly more often in patients with shoulder injuries (82.9% vs. 69.6%). Injuries of the abdomen, pelvis, and lower extremity showed no correlation with shoulder injuries, whereas head and spine injuries showed a significant correlation.

**Conclusion:**

Shoulder injuries are very common in polytraumatized patients. Together with their distinctive concomitant injuries, they have an aggravating impact on the clinical progress. Our data confirm the correlation with thoracic injuries. Furthermore, we identified an increased risk of shoulder injuries in motorbike, bicycle, and pedestrian accidents. An increase in mortality could not be identified.

## Introduction

Severe trauma is the sixth leading cause of death worldwide, and among young adults under 35 years of age, it is the leading cause of death and disability [1]. This changes the awareness from the patient’s survival to recovery, outcome, and quality of life after trauma. In polytraumatized patients, extremity injuries are highly prevalent, occurring in 58.6% of cases [2]. The outcome of polytraumatized patients is worse after additional extremity injuries since they may suffer from severe functional posttraumatic deficits [3–6]. Therefore, the therapy of extremity injuries in multiple injured patients plays a major role in the long-term outcome.

In major trauma, injuries to the shoulder, including severe nerve and vessel injuries, are frequently seen [7] and may represent some of the most common musculoskeletal disorders appearing in the emergency room [8, 9]. In motorbike accidents and polytraumatized patients, upper extremity injuries are frequently seen [2, 10].

In the present literature, several studies highlight the relevance of scapular fractures in a severe trauma or shoulder injuries in general [11–15]. Nevertheless, there are no studies of the TraumaRegister DGU® or similar international databases available that provide a complete overview of injuries of the shoulder in severe multiple trauma and their association with outcome, trauma mechanisms, or concomitant injuries.

The purpose of this study was to investigate the prevalence and clinical features of shoulder injuries in polytraumatized patients. Furthermore, the impact on mortality was assessed, and concomitant injuries were identified.

We presumed that, in the treatment of severely injured patients, the early identification of patients with a high risk for shoulder injuries might help reduce delayed diagnoses and decrease complications. We hypothesized a high prevalence of shoulder injuries in traffic accidents and a high prevalence of concomitant injuries of the thorax leading to an aggravated clinical course and higher ISS.

## Materials and methods

### The TraumaRegister DGU®

The TraumaRegister DGU® (TR-DGU) of the German Trauma Society [Deutsche Gesellschaft für Unfallchirurgie (DGU)] was initiated in 1993. The aim of this multi-center database is to provide a pseudonymized and standardized documentation of severely injured patients. Data are collected prospectively in four consecutive time phases from the site of the accident until discharge from hospital: (a) Pre-hospital phase, (b) emergency room and initial surgery, (c) intensive care unit, and (d) discharge. The documentation includes detailed information on demographics, injury pattern, mechanism of injury, comorbidities, pre- and in-hospital management, a course on intensive care unit, relevant laboratory findings including data on transfusion, length of hospital stay, and outcome of each individual. The inclusion criterion is admission to hospital via an emergency room with subsequent ICU/IMCU care or reaching the hospital with vital signs but dying prior to admission to ICU/IMCU.

The infrastructure for documentation, data management, and data analysis is provided by the Academy for Trauma Surgery (AUC-Akademie der Unfallchirurgie GmbH, München, Germany), an affiliation of the German Trauma Society. The scientific leadership is provided by the Committee on Emergency Medicine, Intensive Care and Trauma Management (Section NIS) of the German Trauma Society. The participating hospitals submitted their data pseudonymized into a central database via a web-based application. Scientific data analysis is approved according to a peer-review procedure established by Section NIS.

The participating hospitals are primarily located in Germany (90%), but an increasing number of hospitals from other countries contribute data as well. Currently, more than 33,000 cases from over 600 hospitals are entered into the database each year.

Participation in TraumaRegister DGU® is voluntary. For hospitals associated with the trauma network of the DGU®, however, the entry of at least a basic data set is obligatory for reasons of quality assurance.

The present study is in line with the publication guidelines of the TraumaRegister DGU® and was registered as TR-DGU project ID 2014–029.

### Study population and inclusion/exclusion criteria

In Germany, 608 participating hospitals contributed their data to the TR-DGU in the dataset from 2002–2013. All patients with an initial ISS ≥ 16 that were documented in the TR-DGU from 2002–2013 were included in the present study. Excluded were all patients with an ISS ≥ 16 due to a single craniocerebral injury (CCI). In sum, 54,076 patients were included in the study. Patients were divided into two subgroups: the “shoulder” group with patients presenting shoulder injuries (15,115 patients), and the “non-shoulder” group which included patients without the presence of any shoulder injuries (38,961 patients). The severity of injuries was documented and coded according to the 2005 revised version of the AIS [16]. The AIS scales the severity of any injury as 1 (minor), 2 (moderate), 3 (severe, but not life threatening), 4 (serious, life threatening), 5 (critical with uncertain survival), and 6 (maximum and currently untreatable) [16]. However, in this study, the severity of the documented injuries only ranges from AIS one to four. Following injuries of the shoulder are represented in this study: fractures, dislocations, amputations, and soft tissue lesions. The shoulder is comprised of bony and ligamentous structures and in this study the shoulder region was defined to include the shoulder joint, acromio-/sterno- clavicular joint, proximal humerus, scapula, clavicle, nerves of the brachial plexus, tendons and vessels. Due to the documentation of the database, injuries to the acromioclaviclular (AC) and sternoclavicular (SC) joint are counted as one lesion. For reasons of simplification injuries to the glenohumeral joint and tendons were summarized as “other”. Further specification of the injuries is limited. Furthermore, concomitant injuries of the shoulder itself in correlation with proximal humerus fractures were assessed (subdivided in the “humerus” group for patients with proximal humerus fractures and “non-humerus” group for patients without proximal humerus fracture).

Concerning the outcome and specification of injury, the average Revised Injury Severity Classification II (RISC II), mean ISS, mortality, trauma mechanisms, and further clinical and epidemiological data (age, gender, red blood cell (RBC) transfusion, Glasgow Come Scale (GCS), and hospital stay including intensive care unit (ICU) and intermediate care unit (IMCU) were assessed. The concomitant injury pattern of polytraumatized patients demonstrates the prevalence of concomitant injuries (any injury of the specified anatomic region with an AIS score ≥ 2) that occurred in conjunction with a shoulder injury.

### Statistics

Differences between the groups were evaluated with Student’s *t* test for continuous data, whereas the Pearsons’ Chi-Square test was used for categorical data. Continuous values are presented as mean ± standard deviation (SD).

A *p* value ≤ 0.05 (two tailed) was considered to be statistically significant. The statistical analyses were performed with SPSS (SPSS 22.0, IBM Inc., Chicago, IL, USA).

## Results

In severe trauma, approximately every third patient suffered a shoulder injury (*n* = 15,115, 27.9%). Patients in the shoulder group had a significantly increased ISS (29.2 ± 11.6 vs. 28.2 ± 11.9, *p* < 0.0001) and were older (47.4 ± 20.2 years vs. 46.4 ± 21.4 years, *p* < 0.0001). As men comprised the majority of all patients in severe trauma, at 72.5% of all cases, gender could not be identified as a risk factor (Table 1).

**Table 1:** Epidemiology of shoulder injuries, clinical course and outcome

	Shoulder	Non-shoulder	Total	*N*	*p* value
No. of patients	15,115	38,961	54,076	54,076	–
Gender (% males)	73.1%	72.3%	72.5%	53,815	= 0.0770
Age (years)	47.4 ± 20.2	46.4 ± 21.4	46.7 ± 21.1	53,825	< 0.0001
ISS	29.2 ± 11.6	28.2 ± 11.9	28.5 ± 11.8	54,076	< 0.0001
No. of diagnosis	7.2 ± 3.2	5.4 ± 2.8	5.9 ± 2.8	54,076	< 0.0001
Hospital stay (days)	26.2 ± 24.6	24.1 ± 25.2	24.7 ± 25.0	53,943	< 0.0001
ICU (days)	12.9 ± 13.0	11.2 ± 13.7	11.7 ± 13.8	49,826	< 0.0001
RISC II	15.5 ± 24.9	17.2 ± 27.1	16.7 ± 26.5	47,074	< 0.0001
Mortality	14.5%	17.4%	16.6%	47,076	< 0.0001
GCS ≤ 8	27.4%	28.3%	28.1%	44,833	= 0.0540
RBC Transfusion	24.0%	21.4%	22.1%	53,644	< 0.0001

*N* No. of patients with available data

**Fig. 1:**
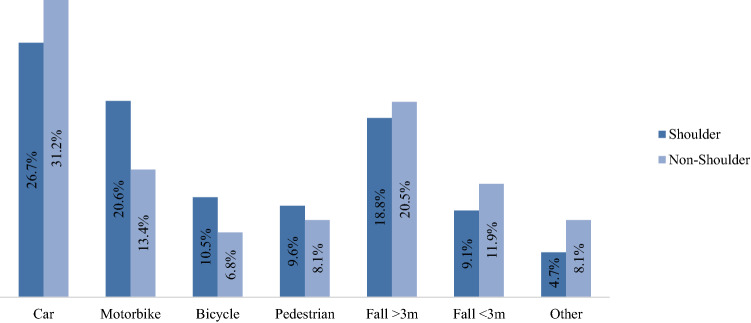
Trauma mechanism (all *p* < 0.0001)

Shoulder injuries were highly associated with blunt trauma in 97.6% of the cases. Moreover, shoulder injuries were caused significantly more often by traffic accidents than by other causes (68.5% vs. 31.5%, *p* < 0.0001), especially in contrast to the non-shoulder group (60.5% vs. 39.5%, *p* < 0.0001). Focusing on traffic accidents, shoulder injuries were caused significantly more often by motorbike (20.6% vs. 13.4%), bicycle (10.5% vs. 6.8%), or pedestrian accidents (9.6% vs. 8.1%) (all *p* < 0.0001). Accidents with cars and trucks (26.7% vs. 31.2%), falls < 3 m (9.1% vs. 11.9%) and falls > 3 m (18.8% vs. 20.5%) were less frequently associated with shoulder injuries (all *p* < 0.0001) (Fig. 1).

**Fig. 2:**
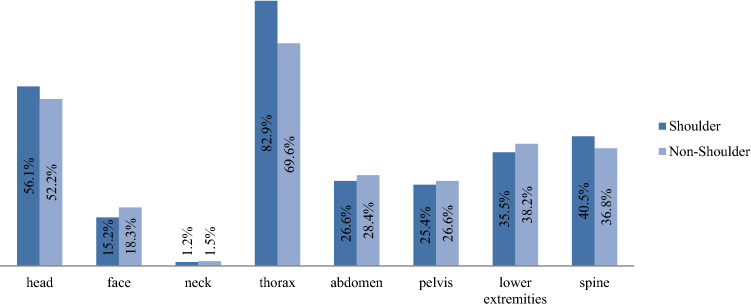
Concomitant injuries of polytraumatized patients (all *p* < 0.0001)

**Fig. 3:**
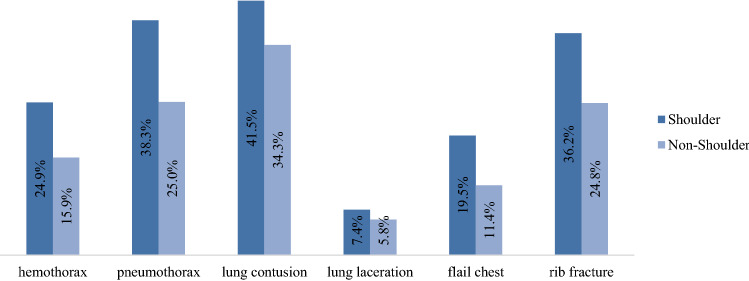
Concomitant thoracic injuries of polytraumatized patients (all *p* < 0.0001)

Concerning the injury pattern of polytraumatized patients and associated concomitant injuries, shoulder injuries were significantly associated with thoracic (82.9% vs. 69.6%), head (56.1% vs. 52.2%), and spine (40.5% vs. 36.8%) injuries (all *p* < 0.0001). Injuries of the shoulder are less common in patients with abdominal (26.6% vs. 28.4%), pelvic (25.4% vs. 26.6%), or lower limb (35.5% vs. 38.2%) trauma (all *p* < 0.0001) (Fig. 2). Highlighting the thoracic injuries, shoulder injuries are associated significantly more often with severe thoracic injuries such as flail-chest, rib fractures, hemothorax, pneumothorax, lung contusion, and lung laceration (Fig. 3).

**Fig. 4:**
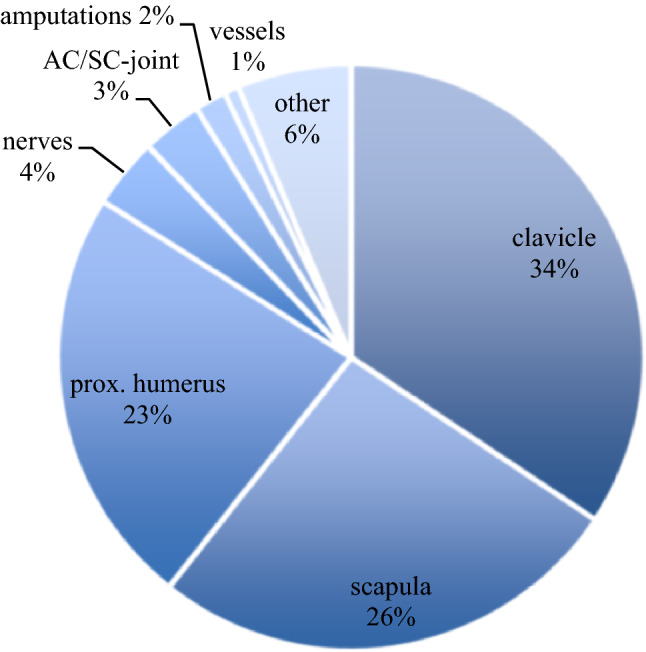
Prevalence of shoulder injuries in polytraumatized patients subdivided into anatomical regions

Analyzing the different entities of shoulder injuries in the shoulder group, the three most often recorded lesions were fractures of the clavicle 34%, scapula 26%, and proximal humerus 23%, followed by injuries of the nerves 4%, AC/SC-joint 3%, amputations 2%, vessel injuries 1%, and other (6%) (Fig. 4). With 83%, fractures make up the majority of shoulder injuries in severe trauma.

**Fig. 5:**
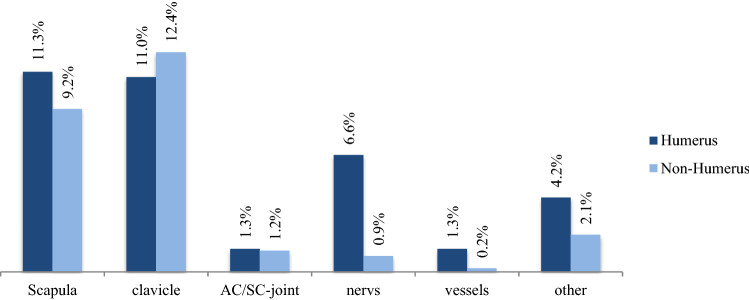
Simultaneous injuries of the shoulder with concomitant proximal humerus fracture (all *p* < 0.0001)

Concomitant injuries of the shoulder itself in the presence of proximal humerus fractures were frequently seen. Injuries to the scapula (11.3% vs. 9.2%), the AC/SC-joint (1.3% vs. 1.2%), nerves (6.6% vs. 0.9%), vessels (1.3% vs. 0.2%), and other (4.2% vs. 2.1%) (all *p* < 0.0001) occurred significantly more often in combination with a proximal humerus fracture. Whereas lesions of the clavicle (11.0% vs. 12.4%) (*p* < 0.0001) occurred less often in combination with a proximal humerus fracture (Fig. 5).

Reviewing and evaluating the outcome of both groups, the patients of the shoulder group spent on average 1.7 days more on ICU/IMCU and 2.1 days more in the acute care hospital (Table 1). On average, patients with shoulder injuries presented 1.7 more diagnoses than patients without shoulder injuries. According to our findings, the vast majority (85.5%) of our study population with shoulder injuries was discharged alive (Table 1).

The mortality rate decreased in the shoulder group (14.5% vs. 17.4%, *p* < 0.0001). In addition, a lower RISC II score was found in patients with shoulder injuries (15.5 ± 24.9 vs. 17.2 ± 27.1, *p* < 0.0001), highlighting the better prognosis of shoulder patients after multiple trauma. Furthermore, shoulder patients showed a more aggravated clinical course than non-shoulder patients, as they had a greater need for red blood cell concentrate (RBC) transfusions (24% vs. 21.4%, *p* < 0.0001) and were more likely to be admitted to the ICU/IMCU (91.7% vs. 93.5%, *p* < 0.0001). Patients in the shoulder group presented a GCS ≤ 8 less often than non-shoulder patients (27.4% vs. 28.3%, p = 0.0543) (all Table 1).

**Fig. 6:**
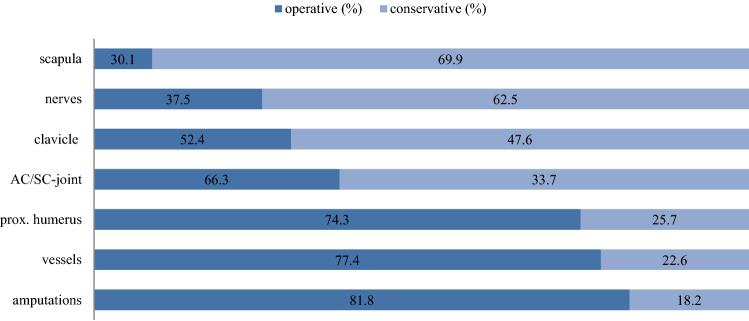
Treatment of shoulder injuries operative vs. conservative

**Fig. 7:**
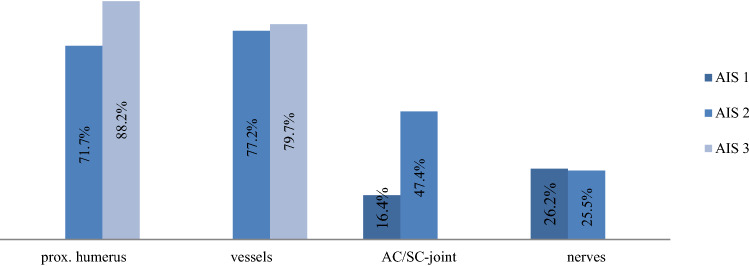
Operation rates depending on the individual severity (classified by AIS) of the individual shoulder injuries (all *p* < 0.0001)

Concerning the operative treatment of lesions in shoulder trauma, our data showed that amputations (81.8%), vessel injuries (77.4%), and prox. humeral fractures (74.3%) were most likely to undergo surgical treatment. Clavicle fractures, nerve lesions, scapula fractures, and injuries to the AC/SC-joint were more often treated conservatively (Fig. 6). The rate of surgical treatment increased significantly with the severity of the injury (according to the documented AIS) (Fig. 7).

## Discussion

Extremity injuries have a high prevalence in severe multiple trauma, with 58.6% of multiple trauma patients presenting significant extremity injury. Affection of the clavicle is the third most common fracture in polytraumatized patients (10.4%), only surpassed by femoral (16.5%) and tibial (12.6%) fractures [2]. Previous studies already investigated lower limb and extremity injuries in severe trauma patients, indicating the need to analyze shoulder injuries in severe trauma [2, 17].

In this context, our findings can be summarized as followed.

Polytraumatized patients (ISS ≥ 16) with shoulder injuries:

- Show a higher rate of head and thoracic injuries.
- Suffer significantly more often from pneumothorax and hemothorax.
- Stay longer on the ICU and in the acute care hospitals.
- Present a higher ISS than severely injured patients without shoulder injuries.
- Were likely to have been involved in a traffic accident.

The high prevalence of shoulder injuries (27.9%) in severely injured patients underlines the importance of understanding the main mechanisms and factors leading to shoulder injuries. Banerjee et al. already showed a 39.6% prevalence of upper extremity injury in polytraumatized patients [2]. Injuries of the shoulder girdle occur more frequently when compared to other upper extremity injuries in severe trauma, such as injuries of the hand (3.5%) [18] and fractures of the radius and ulna (15%) [2]. In our study of 15,115 patients with shoulder injuries, a clavicle fracture occurred most frequently (34%) followed by scapula fractures (26%) and proximal humerus fractures (23%). These findings are similar in some regards to Gottschalk et al. (44.3% clavicle fractures, 25.8% scapula fractures and 16.5% proximal humerus fractures) [7] and in contrast in some regards to Nordqvist et al. (53% proximal humerus fractures, 29% clavicle fractures, 3% scapular fractures) [13] and Veysi et al. (6.8% scapula fractures) [19]. In particular, scapula fractures are associated with high-energy trauma and are often accompanied by severe thoracic (rib fractures, pneumothorax, lung laceration), neurovascular injuries, spinal fractures and pelvic injuries in comparison to patients without scapula fractures [11, 20–22], whereas scapula fractures do not increase the mortality [23]. Concerning concomitant injuries to the shoulder region itself, fractures of the clavicle occur more frequently together with scapula fractures in 25.2% than without the presence of a scapula fracture [11]. In our study, we analyzed concomitant injuries of the shoulder together with proximal humerus fractures that led to increased fractures of the scapula, but decreased fractures of the clavicle. Nevertheless, the deviations in the samples limit the comparability of the studies.

As initially hypothesized, the severity of the injury determines the rate of surgical treatment. Surgical therapy of proximal humerus fractures and injured vessels are very common. In contrast, scapula fractures and clavicle fractures underwent surgical therapy less often. Extraarticular scapula fractures represent approximately two-thirds of all scapula fractures [24] and are commonly treated conservatively [25]. In intraarticular scapula fractures, dislocation, or ipsilateral clavicle fractures (floating shoulder), surgery is the preferred treatment [26, 27]. Previous studies showed a good functional outcome after surgical treatment of complex fractures [28, 29]. The rate of opting for surgical treatment in amputations could be explained due to the “life before limb” strategy in severe trauma [30], as not all amputations received surgery. Unfortunately, detailed follow up of survival of amputated patients, or patients who underwent secondary surgical reconstruction of the amputated limbs could not be generated in the study and, therefore, patients might have died before a possible surgery. Nevertheless, depending on their injury, traumatic amputations and vessel injuries can quickly become life threatening and require immediate surgical treatment. Fractures of the proximal humerus in high-energy trauma are likely to present multiple parts and concomitant soft tissue and neurovascular injuries, leading to primary surgical treatment [31, 32]. A delay in the surgical treatment of proximal humerus fractures should be avoided, as that can lead to increased morbidity, length of hospital stay, and complications such as pneumonia and on-site infections [33]. In multiple injured patients, however, the decision to treat individual lesions surgically follows triage and damage control strategy [34]. This could also delay operative treatment of shoulder injuries. As polytraumatized patients present a variety of injuries, as well as systemic issues up to organ failure, the treatment might not only be decided due to the anatomic issue of the shoulder.

The associated concomitant injuries of shoulder injuries in the shoulder group aggravate the clinical course after severe multiple trauma. They received RBC transfusions, which are known to trigger complications and mortality dose dependence [35], significantly more often. Our findings confirm that shoulder injuries are strongly associated with injuries of the thorax, spine, and head (Figs. 2, 6). Thoracic injuries include the occurrence of pneumothorax, hemothorax, stable and unstable rib fractures, and pulmonary contusion and laceration. In American level 1 trauma centers, admitted patients with shoulder girdle injuries (mean ISS 17.8) presented concomitant thoracic injuries in 36.8%, and head injuries in 31.5% of the cases, respectively [7].

We hypothesize that the high correlation of thoracic injuries with shoulder injuries is caused by the anatomical proximity of these structures and, therefore, the thorax is likely to be simultaneously injured in a shoulder injury, and vice versa. Additionally, in the shoulder group we observed numerous motorbike accidents, which are known to increase the risk of thoracic injuries [36]. In severely injured patients, severe thoracic trauma increases the morbidity and mortality especially due to the increased risk of developing an acute respiratory distress syndrome (ARDS) [3, 37, 38]. Previous studies showed that up to 23.5% of thoracic injuries and 35.7% of scapula fractures are initially overlooked [4, 15]. Therefore, thoracic injuries should always be considered in the occurrence of shoulder injuries. Furthermore, the coexistence of moderate brain injury in combination with extracranial injuries in the polytraumatized patient leads to a doubled rate of mortality [39].

Due to severe concomitant injuries, our findings underline the importance of an early computed tomography scan (CT-scan) of severely traumatized patients, particularly as suggested by guidelines [5]. Additionally, a CT-scan-image-reconstruction (3D reconstruction) or CT-scan of the shoulder girdle is reasonable if there is any clinical or radiological suspicion of scapula fracture [40].

Nevertheless, structured and repeated clinical examinations (body check) remain a key aspect in acute trauma care, especially to identify concomitant injuries that may have been missed initially. Further studies showed that brachial plexus injuries may be present in 1% of multiple trauma patients [41]. With 4%, our data present a significant increase of nerve lesions in shoulder injuries. Injuries of the upper limb with neurological complications such as brachial plexus injuries frequently present severe functional disability in the follow-up and negatively affect the outcome [42, 43]. In a previous study of the Australian trauma registry, a prolonged stay, higher costs, and greater complexity of complications in patients with upper extremity injuries after major trauma were identified [44]. Extremity injuries, especially proximal humerus fractures, can cause long-term movement restrictions. This can lead to a loss of function and quality of life [45].

Focusing on the ISS and RISC II, shoulder patients presented a significantly increased ISS. Unexpectedly, the RISC II score and the mortality of the study population are lower in the shoulder group, and a contemporaneous increase with the ISS could not be shown. Initially, we assumed a higher RISC II and higher mortality in the shoulder group due to the increased ISS. Previous studies already showed a lower mortality rate in severely injured patients with scapular fractures and aggravated concomitant injuries [11, 23], but this remains controversial, as other authors showed an increased ISS and mortality rate in scapula fractures [15]. Nevertheless, the clinical relevance of an increased ISS in the shoulder group seems reasonable in correlation with a significant increase of concomitant thoracic, head, and spine injuries (Figs. 2, 6). In contrast, the non-shoulder group presented more abdominal trauma with a known mortality rate of up to 25% in severe trauma [46]. Blunt thoracic trauma only requires surgery in 10% of the cases [47]. Furthermore, an increased ISS followed by decreased mortality rate was observed in comparable studies on extremity and joint injuries [2, 17].

With regard to the higher ISS in the shoulder group, a previous study showed that a higher ISS could predict lower general health condition with respect to the long-term outcome after trauma [48]. However, it must be mentioned that the ISS has limitations in predicting the severity of the trauma as it does not represent physiological variables indicating shock symptoms, and multiple injuries in the same body region are not represented [49].

Regarding the mechanism of injury, traffic injuries have a huge impact on trauma cases worldwide and are the leading cause of death of young adults in Europe [50, 51]. This relevance is confirmed by our study, as the majority of our study population suffered from traffic accidents. Other national and international studies illustrate and support our findings, identifying traffic injuries as highly predictive for a traumatic affection of extremities, in particular upper extremity injuries [2, 52]. Furthermore, motorbike, bicycle, and pedestrian accidents have the highest incidence in our study population. Another study on motorbike accidents underlines our findings as motorbike accidents presented upper extremity injuries in 35% of their cases. Injuries of the shoulder girdle represent the largest group of upper extremity injuries, at 57% [10]. We assume that the unprotected exposure of the shoulder in motorbike, pedestrian, and bicycle accidents might be the main risk factor contributing to the significantly increased prevalence of shoulder injuries in these cases. In comparison, car and truck accidents and falls more often result in lower limb injuries [17, 53]. In car accidents, the so-called dashboard injury leads to major trauma of the lower extremity. The shoulder girdle is more protected in these injuries and, therefore, not frequently affected [17].

## Limitations

The present study has limitations, as the analyzed data is retrospective. As the participating hospitals have different levels of patient-centered care, the treatment after trauma could differ. As shoulder injuries could have been initially overlooked, there is a chance that shoulder injuries have been under reported. Furthermore, in polytraumatized patients, minor injuries may be omitted. The choice of diagnostics in the database leading to the diagnosis is not reported, so the accuracy of the data database is dependent on the individual examiner and submission of data in the register. Unfortunately, the exact specification of soft tissue injuries could not be classified.

## Conclusion

In summary, our findings show that shoulder injuries in severely injured patients are frequently associated with severe concomitant injuries, mainly of the chest, head, and spine. High-speed traffic accidents are the most common injury mechanisms, particularly in motorbike, bicycle, and pedestrian accidents. Previous studies on extremity injuries of the TraumaRegister DGU® confirmed the increase of the ISS, duration of stay, and aggravated clinical course of extremity injuries [2, 17].

Shoulder injuries have an aggravating impact on the clinical course and prolong the clinical stay. As multiple trauma patients with shoulder injury present more diagnoses, the clinical management and treatment of the patient are aggravated. This is demonstrated by a higher initial ISS and longer stay on ICU/IMCU. Nevertheless, an increased rate of mortality in patients with shoulder injuries could not be identified. Our results correspond with empirical data in our daily clinical routine and other national and international findings in the literature. Furthermore, because of the large sample size of the analyzed study population and the high number of included trauma centers providing data, the clinical relevance of our findings seems significant. In the emergency room (ER) and in the tertiary survey, potential shoulder injuries and identified concomitant injuries should be closely considered.
